# Association between plasma sRAGE and emphysema according to the genotypes of AGER gene

**DOI:** 10.1186/s12890-022-01848-9

**Published:** 2022-02-10

**Authors:** Sooim Sin, Myung-nam Lim, Jeeyoung Kim, So Hyeon Bak, Woo Jin Kim

**Affiliations:** 1 Department of Internal Medicine, School of Medicine, Kangwon National University Hospital, Kangwon National University, Chuncheon, 24341 Republic of Korea; 2 Department of Internal Medicine and Environmental Health Center, School of Medicine, Kangwon National University Hospital, Kangwon National University, Chuncheon, Republic of Korea; 3 Department of Radiology, , School of Medicine, Kangwon National University Hospital, Kangwon National University, Chuncheon, Republic of Korea

**Keywords:** Glycation end-product, Advanced, Receptor for advanced glycation end products, Pulmonary disease, Chronic obstruction, Pulmonary emphysema, Soluble receptor for advanced glycation end product

## Abstract

**Background:**

Higher soluble receptor for advanced glycation end product (sRAGE) levels are considered to be associated with severe emphysema. However, the relationship remains uncertain when the advanced glycation end-product specific receptor (AGER) gene is involved. We aimed to analyse the association between sRAGE levels and emphysema according to the genotypes of rs2070600 in the AGER gene.

**Methods:**

We genotyped rs2070600 and measured the plasma concentration of sRAGE in each participant. Emphysema was quantified based on the chest computed tomography findings. We compared sRAGE levels based on the presence or absence and severity of emphysema in each genotype. Multiple logistic and linear regression models were used for the analyses.

**Results:**

A total of 436 participants were included in the study. Among them, 64.2% had chronic obstructive pulmonary disease and 34.2% had emphysema. Among the CC-genotyped participants, the sRAGE level was significantly higher in participants without emphysema than in those with emphysema (*P* < 0.001). In addition, sRAGE levels were negatively correlated with emphysema severity in CC-genotyped patients (r = − 0.268 *P* < 0.001). Multiple regression analysis revealed that sRAGE was an independent protective factor for the presence of emphysema (adjusted odds ratio, 0.24; 95% confidence interval (CI) 0.11–0.51) and severity of emphysema (β = − 3.28, 95% CI − 4.86 to − 1.70) in CC-genotyped participants.

**Conclusion:**

Plasma sRAGE might be a biomarker with a protective effect on emphysema among CC-genotyped patients of rs2070600 on the AGER gene. This is important in determining the target group for the future prediction and treatment of emphysema.

**Supplementary Information:**

The online version contains supplementary material available at 10.1186/s12890-022-01848-9.

## Background

Chronic obstructive lung disease (COPD), a major cause of death worldwide, is characterised by chronic airflow limitation due to small airway disease and emphysema [1]. In addition to its association with COPD, emphysema is an independent predictor of mortality [2, 3]. Consequently, soluble receptor for advanced glycation end product (sRAGE) levels have gained prominence as biomarkers of emphysema. Several studies have shown that low levels of sRAGE are associated with emphysema and severe emphysema [4–6]. In addition, progression of emphysema is related to sRAGE levels [7].

RAGE, a multiligand transmembrane receptor, is highly expressed in normal lung tissues and regulates inflammatory pathways [8]. Chronic inflammatory diseases, including diabetes mellitus, arthritis, and cardiovascular diseases, are associated with the RAGE pathway [9–11]. Likewise, a number of studies have demonstrated a relationship between RAGE and emphysema [12–14]. Furthermore, genome-wide association studies (GWASs) have shown that the RAGE gene for the advanced glycation end-product specific receptor, *AGER*, which generates RAGE and consequently sRAGE, is associated with either COPD or emphysema [15, 16]. Among the multiple variants of *AGER*, rs2070600 is a particularly notable sentinel variant related to emphysema. The minor allele of rs2070600 was identified to be associated with better lung function, low risk of COPD, decreased quantification of emphysema, and low sRAGE levels.

Thus, these findings suggest that high levels of sRAGE have a protective effect on the risk of emphysema. However, the minor allele of rs2070600 is simultaneously related to low levels of sRAGE and a decreased risk of emphysema. This study sought to evaluate the relationship between sRAGE and emphysema according to the rs2070600 polymorphism. We hypothesised that the association between sRAGE and emphysema is different for each rs2070600 polymorphism.

## Methods

### Study population

**Fig. 1:**
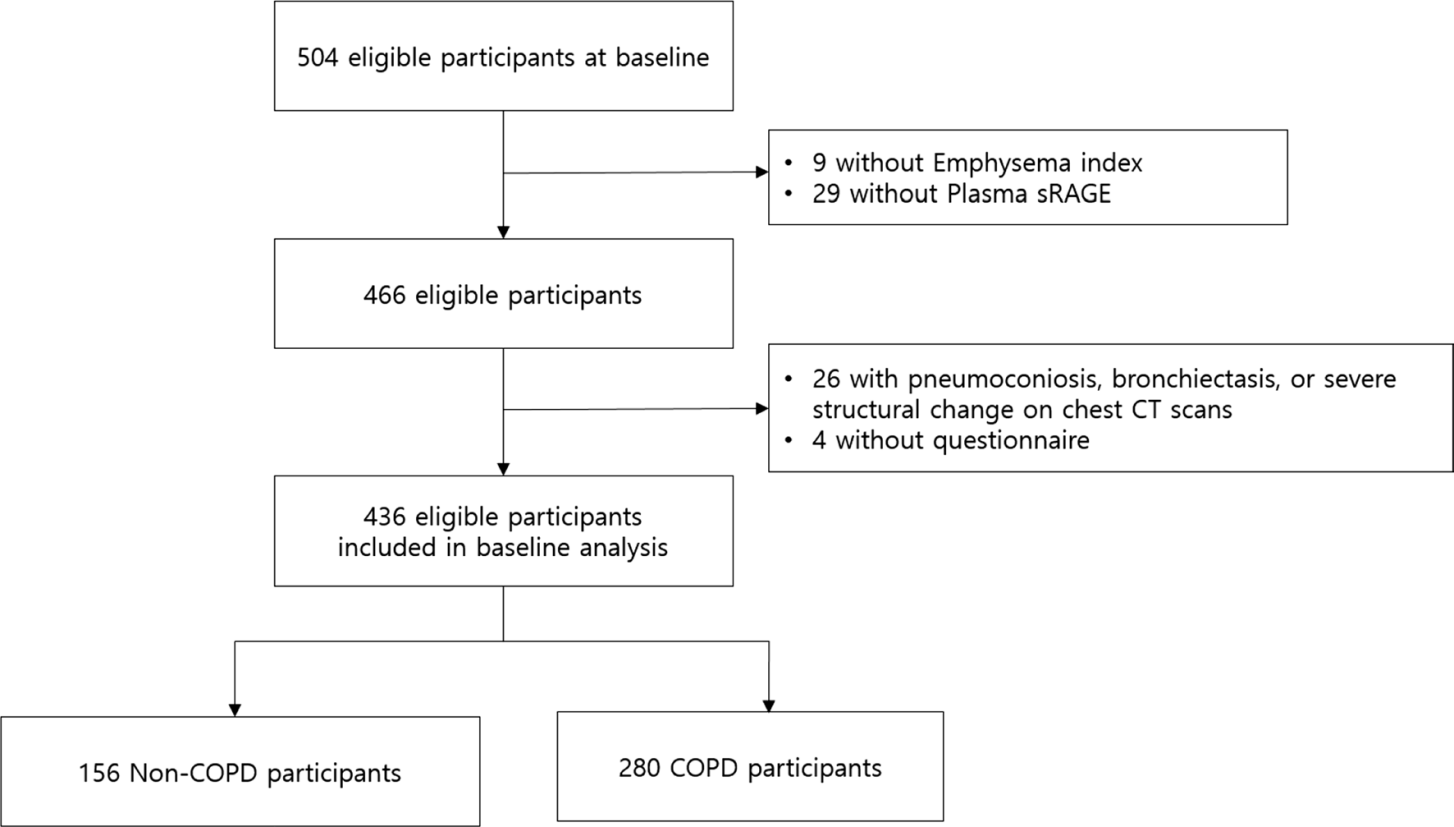
Flow chart of the study

A total of 504 participants from the COPD in the dusty area (CODA) cohort were enrolled from January 2012 to December 2017 (Fig. 1). CODA is a prospective cohort composed of patients with airflow limitation and healthy volunteers residing in the Kangwon and Chungbuk provinces of South Korea. The details of the cohorts have been described previously [17, 18]. Clinical information, including medical history, subjective symptoms, and lung function results, was assessed by medical interviews, questionnaires, physical examinations, and spirometry. Patients with a post-bronchodilator forced expiratory volume in 1 s (FEV_1_) to forced vital capacity (FVC) ratio below 0.7 at baseline were defined as having COPD [1]. Patients with COPD were divided into stages 1 to 4 according to airflow limitation based on the Global Initiative for Chronic Obstructive Lung Disease (GOLD) guidelines [1].

Written informed consent was obtained from all participants. This study was approved by the Institutional Review Board of the Kangwon National University Hospital (IRB No. KNUH 2012-06-007).

### Emphysema quantification

All participants underwent volumetric thin-section chest computed tomography (CT at full inspiration in the supine position. CT images were obtained using a dual-source CT scanner (Somatom Definition; Siemens Healthcare, Forchheim, Germany). Quantification was performed using the Aview® system (Coreline Soft Inc.). We quantified emphysema which was defined as lung parenchyma below the -950 Hounsfield unit threshold on CT images [19, 20]. The quantified emphysema was used as a continuous variable and the emphysema index. In addition, as a categorical variable, the presence or absence of emphysema was based on an emphysema index of 5% of the total lung area, as defined in previous studies [21, 22]. The detailed methodology of the CT protocols has been previously published [23].

### Measurement of plasma sRAGE levels

Plasma samples were obtained from the participants to quantify sRAGE levels using a commercially available enzyme-linked immunosorbent assay (ELISA) (human RAGE Quantikine® ELISA Kit SRG00; R&D System, Minneapolis, MN, USA), according to the manufacturer’s instructions. The ELISA plates were read using a microplate reader (Molecular Devices, Sunnyvale, CA, USA).

### Genotyping of rs2070600

Genotyping was performed using blood samples from the Asian Precision Medicine Research Array (APMRA, Afymetrix, CA, USA). DNA samples were hybridised to an array in the GeneTitan MC Instrument (Afymetrix) according to the GeneTitan® Multichannel Instrument User’s Manual, using the Axiom APMRA 96-ARRAY. After ligation, the arrays were stained and imaged using a GeneTitan MC instrument (Afymetrix).

### Statistical analysis

Data were analysed using the chi-square test for categorical variables and Student’s t-test for continuous variables. The relationships between the two continuous parameters were evaluated using the Pearson’s correlation coefficients. Multiple logistic and linear regression analyses were performed to adjust for covariates which were clinically important or statistically significant in the univariate analyses. Log-transformed sRAGE was used for the statistical analysis. Statistical significance was defined as a two-sided *P-value *< 0.05. Statistical analyses were conducted using SAS (version 9.4; SAS Institute, Cary, NC, USA) and R (version 3.2.3 (The R Foundation for Statistical Computing, Vienna, Austria, http://www.Rproject.org).

## Results

### Demographics of the study population

Among the 504 eligible participants, 436 were included in the analysis (Fig. 1). The baseline demographics of the study participants are shown in Table 1. The mean age of the participants was 72.3 years, and 316 (72.5%) were male. The number of COPD patients was 280 (64.2%), and among the COPD patients, 143 (51.1%), 118 (42.1%), and 19 (6.8%) were classified into stages 1, 2, and 3, respectively. Overall, 146 (33.5%) participants had either TC or TT genotypes at rs2070600, and 149 (34.2%) participants had emphysema (emphysema index > 6%) on chest CT.

**Table 1:** Baseline demographics and comparison of demographics according to genotypes

	Total N = 436	CC* n = 290	TC + TT* n = 146	*P*
Age, years	72.3 ± 7.1	72.4 ± 7.1	72.2 ± 7.2	0.710
Sex, male, n (%)	316 (72.5)	218 (75.2)	98 (67.1)	0.075
Emphysema index	5.6 ± 6.3	6.5 ± 0.3	4.7 ± 0.5	0.002
Presence of emphysema				0.020
Emphysema (−)	287 (65.8)	180 (62.1)	107 (73.3)	
Emphysema (+)	149 (34.2)	110 (37.9)	39 (26.7)	
COPD, n (%)				0.040
Non-COPD	156 (35.8)	93 (32.0)	63 (43.2)	
GOLD Stage 1	20 (4.6)	12 (4.1)	8 (5.5)	
GOLD Stage 2	143 (32.8)	92 (31.7)	51 (34.9)	
GOLD Stage 3	118 (27.0)	90 (31.0)	28 (18.2)	
Smoking status, n (%)				0.046
Current	96 (22.1)	72 (24.9)	24 (16.5)	
Former	180 (41.5)	122 (42.2)	58 (40.0)	
Never	158 (36.4)	95 (32.9)	63 (43.5)	
Smoking, Pack-years	17.1 ± 22.8	18.1 ± 22.4	15.2 ± 23.6	0.223
Education				0.304
< Elementary school	132 (31.1)	90 (31.9)	42 (29.4)	
Elementary school	164 (38.6)	101 (35.8)	63 (44.0)	
Middle school	63 (14.8)	38 (13.5)	25 (17.5)	
≥ High school	66 (15.5)	53 (18.8)	13 (9.1)	
sRAGE, pg/mL	666.7 ± 338.8	720.9 ± 341.4	558.9 ± 307.5	< 0.001
Log sRAGE	6.4 ± 0.4	6.4 ± 0.4	6.2 ± 0.5	< 0.001

Categorical variables are expressed as number (%) and continuous variables are expressed as mean ± standard deviation

*COPD* chronic obstructive pulmonary disease, *sRAGE* soluble receptor for advanced glycation end products

*Genotypes of rs2060700

No significant differences were observed in the mean age or sex distribution according to the different polymorphisms of rs2070600. Participants with the CC genotype had more severe emphysema and COPD than those with the T allele (Table 1). The log-transformed sRAGE levels followed a normal distribution (Additional file 1: Fig. 1).

### Emphysema status according to plasma sRAGE level in each genotype

**Fig. 2:**
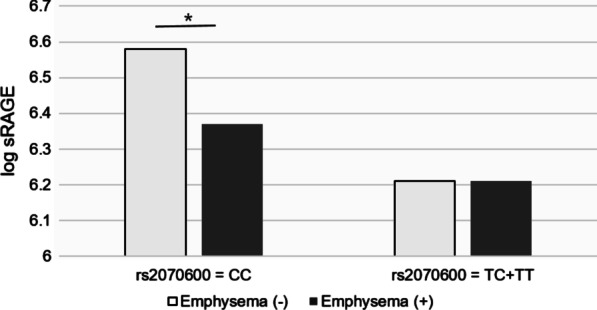
Emphysema status according to plasma sRAGE level in each genotype

We grouped the participants according to the rs2070600 polymorphism. Plasma sRAGE levels in the presence or absence of emphysema were compared in each genotype group. The sRAGE level was significantly higher in participants without emphysema than in those with emphysema in all participants (*P* = 0.006). Similarly, in the CC genotyped group, sRAGE levels were significantly higher in participants without emphysema than in those with emphysema (*P* < 0.001). However, there was no difference in sRAGE levels between the participants with and without emphysema among the T allele-containing genotype group (Table 2, Fig. 2).

**Table 2:** Emphysema status according to plasma sRAGE level in each genotype

	Total		CC*		TC + TT*	
	sRAGE	*P*	sRAGE	*P*	sRAGE	*P*
Emphysema (−)	6.44 ± 0.47		6.58 ± 0.41		6.21 ± 0.48	
Emphysema ( +)	6.32 ± 0.40	0.006	6.37 ± 0.38	< 0.001	6.21 ± 0.45	0.976

Levels of sRAGE are log transformed and expressed as mean ± standard deviation

*sRAGE* soluble receptor for advanced glycation end product

*Genotypes of rs2060700

### Correlation between emphysema index and sRAGE

**Fig. 3:**
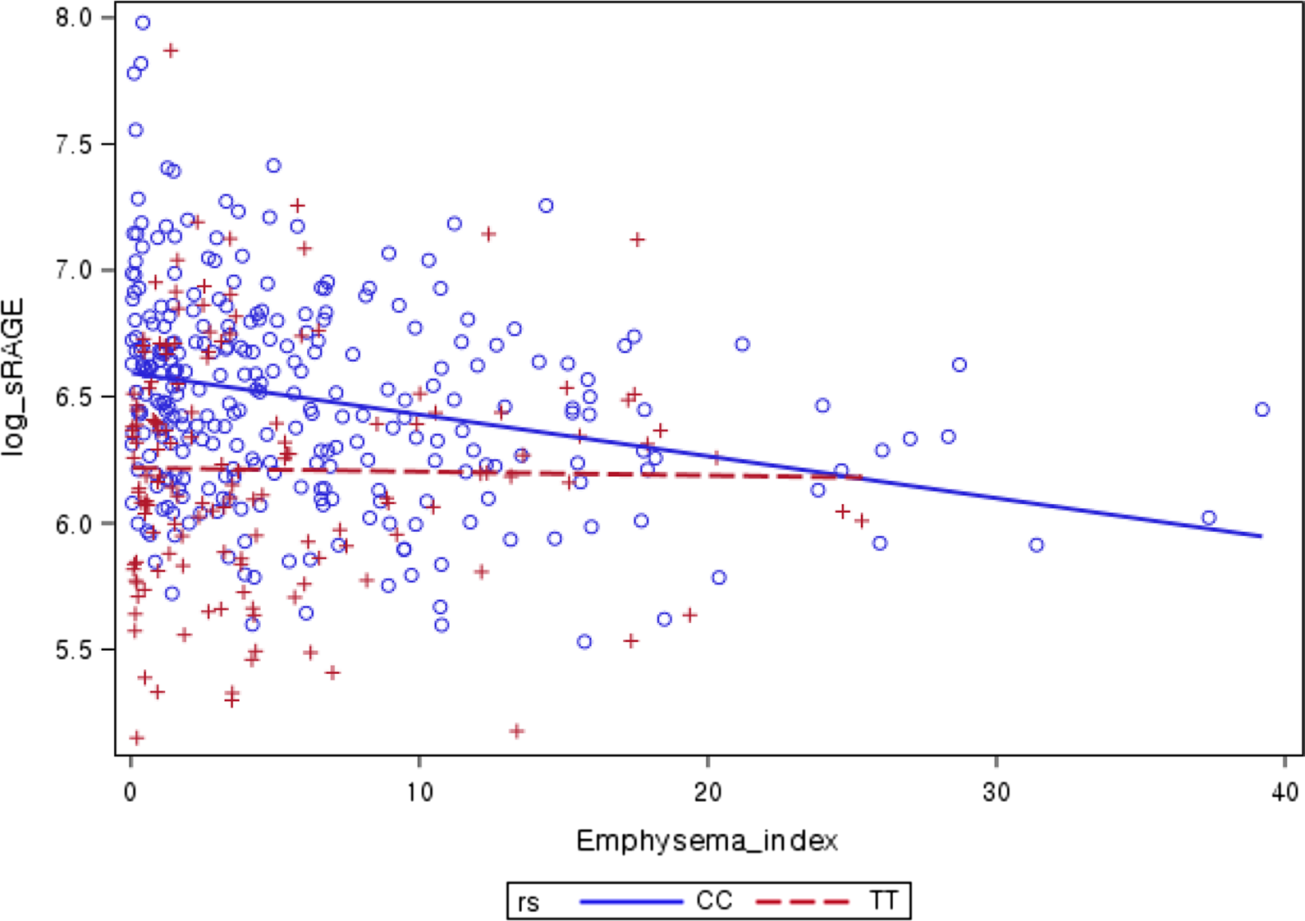
Scatter plot presenting correlation between sRAGE and lung function or emphysema in each genotype. CC genotype group, r = − 0.268 and *P* < 0.001; TC + TT genotype group, r = − 0.017 and *P* = 0.836

In the correlation analysis, the sRAGE level was negatively correlated with the emphysema index in all participants (r = − 0.146, *P* = 0.002). Interestingly, when we divided the participants according to genotype, this correlation was only significant in CC-genotyped participants (r = − 0.268, *P* < 0.001). In the T-allele-containing genotype group, there was no clear correlation between sRAGE and emphysema (Table 3). This correlation was also observed in the scatter plot (Fig. 3).

**Table 3:** Correlation between sRAGE and lung function or emphysema in each genotype

	Total N = 436		CC* n = 290		TC + TT* n = 146	
	Log sRAGE					
	r	*P*	r	*P*	r	*P*
Post-BD FVC (L)	−0.034	0.478	−0.059	0.314	−0.021	0.800
Post-BD FEV_1_ (L)	−0.004	0.931	0.011	0.848	−0.001	0.990
Post-BD FEV_1_/FVC (%)	0.034	0.484	0.118	0.045	0.003	0.968
Emphysema index	−0.146	0.002	−0.268	< 0.001	−0.017	0.836

*sRAGE* soluble receptor for advanced glycation end product, *BD* bronchodilator, *FVC* forced vital capacity, *FEV*_*1*_ forced expiratory volume in 1s

*Genotypes of rs2060700

### Multiple regression analyses for emphysema

To identify the risk factors, logistic regression analyses for the risk of emphysema were conducted for each genotype group. In the multiple logistic model, male sex and COPD were risk factors for emphysema, and sRAGE was a protective factor for emphysema in CC genotyped participants (aOR for log sRAGE 0.24; 95% CI, 0.11–0.51), whereas sRAGE was not significantly associated with the T allele (Table 4). In addition, linear regression analyses for the severity of emphysema were conducted for each genotype group. Multiple linear regression analysis showed that the level of sRAGE had a negative correlation with the emphysema index in CC genotyped participants (β = − 3.28; 95% CI, − 4.86 to − 1.70), while the level of sRAGE showed no relationship in participants with the T allele (Table 5).

**Table 4:** Logistic regression model for emphysema

		Univariable	Multivariable
		OR (95% CI)	aOR (95% CI)
**rs2070600 = CC**			
Age		1.02 (0.98–1.05)	1.00 (0.96–1.05)
Sex	Male vs. Female (ref.)	10.04 (4.18–24.12)	5.40 (1.53–19.11)
Smoking status	Current vs. None (ref.)	5.85 (2.73–12.55)	0.85 (0.27–2.62)
	Former vs. None (ref.)	7.63 (3.78–15.40)	2.03 (0.73–5.62)
Income	≤ 49 vs. ≥ 100 (ref.)	1.33 (0.72–2.45)	
	50–99 vs. ≥ 100 (ref.)	1.74 (0.77–3.89)	
Education	< Elementary vs. high (ref.)	0.38 (0.19–0.77)	
	Elementary vs. high (ref.)	0.59 (0.30–1.15)	
	Middle vs. high (ref.)	0.46 (0.20–1.10)	
BMI		0.79 (0.72–0.87)	0.79 (0.70–0.88)
COPD	Yes vs. No (ref.)	4.39 (2.39–8.06)	2.97 (1.53–5.79)
Diabetes	Yes vs. No (ref.)	0.65 (0.31–1.38)	
Kidney disease	Yes vs. No (ref.)	0.01 (0.001–999.9)	
CVD*	Yes vs. No (ref.)	0.71 (0.31–1.61)	
Log sRAGE		0.23 (0.12–0.44)	0.24 (0.11–0.51)
**rs2070600 = TC + TT**			
Age		1.01 (0.96–1.06)	1.02 (0.95–1.09)
Sex	Male vs. Female (ref.)	10.00 (0.01–35.82)	5.11 (0.01–21.99)
Smoking status	Current vs. None (ref.)	10.54 (2.88–38.57)	1.54 (0.33–7.14)
	Former vs. None (ref.)	10.41 (3.33–32.54)	1.65 (0.42–6.41)
Income	≤ 49 vs. ≥ 100 (ref.)	0.38 (0.15–0.97)	
	50–99 vs. ≥ 100 (ref.)	0.46 (0.14–1.52)	
Education	< Elementary vs. high (ref.)	0.10 (0.03–0.36)	
	Elementary vs. high (ref.)	0.29 (0.11–0.77)	
	Middle vs. high (ref.)	0.79 (0.21–3.03)	
BMI		0.77 (0.67–0.88)	0.77 (0.65–0.91)
COPD	Yes vs. No (ref.)	6.27 (2.43–16.20)	4.46 (1.58–12.58)
Diabetes	Yes vs. No (ref.)	1.01 (0.41–2.51)	
Kidney disease	Yes vs. No (ref.)	4.00 (0.85–18.88)	
CVD	Yes vs. No (ref.)	0.47 (0.15–1.47)	
Log sRAGE		0.77 (0.35–1.70)	0.61 (0.23–1.61)

OR, odds ratio; BMI, body mass index; COPD, chronic obstructive pulmonary disease; CVD, cardiovascular disease; sRAGE, soluble receptor for advanced glycation end product

**Table 5:** Linear regression model for severity of emphysema

		Univariable	Multivariable
		beta (95% CI)	beta (95% CI)
**rs2070600 = CC**			
Age		0.09 (−0.02–0.20)	0.03 (−0.06–0.13)
Sex	Male vs. Female (ref.)	5.42 (3.74–7.09)	1.90 (−0.55–4.35)
Smoking status	Current vs. None (ref.)	5.15 (3.26–7.05)	0.81 (−1.77–3.40)
	Former vs. None (ref.)	5.78 (4.13–7.44)	2.81 (0.45–5.17)
Income	≤ 49 vs. ≥ 100 (ref.)	0.64 (−1.24–2.52)	
	50–99 vs. ≥ 100 (ref.)	2.56 (0.02–5.10)	
Education	< Elementary vs. high (ref.)	−3.11 (−5.29 to − 0.93)	
	Elementary vs. high (ref.)	−1.68 (−3.81–0.46)	
	Middle vs. high (ref.)	−1.82 (−4.50–0.86)	
BMI		−0.81 (−1.06 to − 0.56)	−0.63 (−0.87 to − 0.38)
COPD	Yes vs. No (ref.)	4.78 (3.21–6.34)	2.87 (1.4–4.31)
Diabetes	Yes vs. No (ref.)	−1.93 (−4.15–0.30)	
Kidney disease	Yes vs. No (ref.)	−3.29 (−10.66–4.07)	
CVD*	Yes vs. No (ref.)	−1.74 (−4.22–0.75)	
Log sRAGE		−4.37 (−619 to − 2.55)	−3.28 (−4.86 to − 1.70)
**rs2070600 = TC + TT**			
Age		0.04 (−0.09–0.16)	0.002 (−0.10–0.11)
Sex	Male vs. Female (ref.)	5.14 (3.39–6.89)	1.94 (−0.58–4.46)
Smoking status	Current vs. None (ref.)	3.43 (1.09–5.77)	0.50 (−2.34–3.34)
	Former vs. None (ref.)	5.28 (3.50–7.05)	2.44 (−0.02–4.91)
Income	≤ 49 vs. ≥ 100 (ref.)	−1.53 (−3.97–0.90)	
	50–99 vs. ≥ 100 (ref.)	−1.56 (−4.66–1.53)	
Education	< Elementary vs. high (ref.)	−4.34 (−7.73 to − 0.94)	
	Elementary vs. high (ref.)	−2.96 (−6.21–0.30)	
	Middle vs. high (ref.)	−1.83 (−5.49–1.83)	
BMI		−0.57 (−0.81 to − 0.34)	−0.43 (−0.65 to − 0.22)
COPD	Yes vs. No (ref.)	4.15 (2.44–5.86)	2.57 (1.00–4.15)
Diabetes	Yes vs. No (ref.)	−0.34 (−2.58–1.90)	
Kidney disease	Yes vs. No (ref.)	3.62 (−0.56–7.80)	
CVD	Yes vs. No (ref.)	−2.16 (−4.54–0.21)	
Log sRAGE		−0.20 (−2.15–1.75)	−1.15 (−2.76–0.45)

*OR* odds ratio, *BMI* body mass index, *COPD* chronic obstructive pulmonary disease, *CVD* cardiovascular disease; *sRAGE* soluble receptor for advanced glycation end product

## Discussion

The principal finding of our study was that the rs2070600 polymorphism affected the relationship between emphysema and plasma sRAGE. In participants with the CC allele of rs2070600, plasma sRAGE had a protective effect on emphysema, whereas this effect was not observed in participants with the T allele. Moreover, in participants with the CC allele of rs2070600, the level of plasma sRAGE was negatively correlated with emphysema severity, whereas this correlation was not observed in those with the T allele.

RAGE is a receptor implicated in the mediation and amplification of proinflammatory responses [24]. Pulmonary inflammation through RAGE induces emphysematous changes in animal models [25, 26]. As a soluble extracellular decoy receptor for RAGE, sRAGE protects cells against inflammation and tissue injury. Inhibition of RAGE by sRAGE reduces acute lung injury in an animal model [27].

A number of studies have shown that sRAGE levels are not only decreased in patients with emphysema, but also negatively correlated with the severity of emphysema [4, 6, 7]. Moreover, the progression of emphysema is associated with sRAGE [7]. These studies were based on large populations and validated cohorts such as the Treatment of Emphysema with a Selective Retinoid Agonist (TESRA), Evaluation of COPD Longitudinally to Identify Predictive Surrogate Endpoints (ECLIPSE), and COPDGene cohorts. The results of our study are consistent with those of previous studies. We confirmed that sRAGE levels were significantly lower in participants with emphysema and showed a significant negative relationship with the severity of emphysema. This is the first time that the relationship has been proven in an Asian population. In terms of genetics, the plasma levels of sRAGE were greater in participants with the CC allele than in those with the T allele of rs2070600 [21, 28]. This finding has also been identified in various populations, including Koreans [29]. In this study, we confirmed this relationship between the allele of rs2070600 and levels of sRAGE. In addition, previous GWASs have identified that the T allele of rs2070600 is associated with a lower risk of emphysema [15, 30]. Our results show that a lower proportion of patients with emphysema and a lower emphysema index in the T allele group also supports previous findings. Combining these proven associations, the paradoxical effect of the T allele of rs2070600 remains an unsolved problem. The minor allele (T) of rs2070600 was associated with a lower risk of emphysema and was simultaneously associated with lower sRAGE levels, which are linked to a high risk of emphysema. In our study, the results of the statistical analysis after grouping the subjects according to the genotype of rs2060700 presented novel insights. Contrary to the statistically significant trends in the overall population, the relationship between sRAGE and emphysema in a minority of the participants, the minor allele group, was not significant. The relationship between sRAGE and emphysema, which was definitely different in the minority group, may have been considered the same as the relationship identified in the majority due to the small number of the minor group in previous cohort studies.

Although GWAS revealed the association between *AGER* gene polymorphisms and emphysema, the underlying pathogenic mechanism is unknown. The RAGE axis is difficult to explain simply because the *AGER* gene affects both RAGE and sRAGE, and simultaneously, sRAGE also affects signalling through RAGE. Here, we suggest the following hypothesis: the *AGER* gene may be involved in the function of RAGE, as well as the concentration of sRAGE. For example, as in previous functional studies on RAGE, the binding affinity of sRAGE to RAGE may differ among alleles. The high binding affinity of sRAGE to RAGE in the participants with the T allele effectively blocked inflammatory signals through RAGE and prevented the development and progression of emphysema despite the low level of sRAGE in the participants with the T allele. In contrast, a low binding affinity would result in various degrees of RAGE blockade depending on the sRAGE level. Additional experimental research is needed to elucidate the pathogenic mechanisms associated with *AGER*.

The study by Pratte et al. also showed significant differences in the level of sRAGE according to genotype of rs2070600, and the level was higher in the CC genotype than in the minor allele genotypes which is in line with our study results [31]. Distinct from the study which focused on the interaction of genotype on the correlation of sRAGE and emphysema, we focused on the individual correlation between sRAGE and emphysema in each genotype. Therefore, our results suggest that the unique and significant association between sRAGE and emphysema in the CC genotype may have clinical value which should not be overlooked. The p value for the interaction of rs2070600 genotype on the correlation between sRAGE and emphysema failed to achieve statistical significance in our study. However, there are limitations in interpreting the results of the interaction p-value, considering the fact that the association between sRAGE and emphysema in the minor allele genotype in our study was not significant.

Our study had several strengths. This was the first study to simultaneously elucidate the associations among emphysema, sRAGE, and polymorphisms of the *AGER* gene. Although robust associations have been identified between pairs of these three factors, contradictions have emerged when emphysema and sRAGE are simultaneously linked with *AGER* genetics. Our study suggests a potential solution for understanding the association between sRAGE and emphysema in relation to *AGER* genetics. Furthermore, to our knowledge, no previous study has assessed sRAGE and emphysema in an Asian population.

There are several limitations to the current study. First, owing to the study design, it was impossible to assess temporal relationships among the factors. Further studies are needed to prove the temporal relationship between the *AGER* gene, sRAGE, and emphysema. Second, there was no difference in sRAGE levels between COPD and non-COPD patients, unlike the findings presented in several studies [4, 32, 33]. Since the studies showed that sRAGE levels are associated with not only the presence of COPD but also the severity of COPD, this might be due to the milder COPD in the majority of our study participants compared to the previous studies. In line with previous studies, our results show that the T allele of rs2070600 is associated with a lower risk of COPD and mild COPD [34–36]. The distribution of the rs2070600 polymorphism was considerably different from that reported in studies of other ethnicities [37, 38]. However, this is in line with previous Asian studies, including studies on the Korean population, and the distribution of *AGER* polymorphisms is well known to differ between ethnic groups [25, 39, 40]. This makes it difficult to apply the results to other ethnicities, and further studies are required for validation in other populations. In statistical analysis, multiple comparisons problem which is made in univariable regression analyses can be an another limitation. Finally, since these results are the first identified results, external replications are needed in larger and different cohorts.

## Conclusion

In conclusion, our study showed that the protective effect of plasma sRAGE on emphysema is valid in participants with the CC genotype of rs2070600 on the *AGER* gene. These findings will provide information about future target groups for the prediction and treatment of emphysema.

## Supplementary Information

**Additional file 1.** **Fig. 1.** Distribution of plasma sRAGE level and log-transformed sRAGE level.

## Data Availability

The datasets used and/or analysed during the current study are available from the corresponding author upon reasonable request.

## Abbreviations

AGER: Advanced glycation end-product specific receptor
BMI: Body mass index
CI: Confidence interval
COPD: Chronic obstructive pulmonary disease
CT: Computed tomography
ELISA: Enzyme-linked immunosorbent assay
FEV_1_: Forced expiratory volume in one second
FVC: Forced vital capacity
GOLD: Global initiative on obstructive lung disease
GWAS: Genome-wide association studies
sRAGE: Soluble receptor for advanced glycation end product
